# Rhomboid flap: Indications, applications, techniques and results. A comprehensive review

**DOI:** 10.1016/j.amsu.2021.102544

**Published:** 2021-07-07

**Authors:** Ajaipal S. Kang, Kevin S. Kang

**Affiliations:** a Department of Surgery and Chief of Plastic Surgery, UPMC Hamot, Erie, PA, 16507, USA; b Geisel Dartmouth Medical School, Hanover, NH, 03755, USA

**Keywords:** Rhomboid flap, Limberg flap: reconstruction, Skin cancer, Cutaneous defect, Transposition, Local flap, RSTL, Relaxed Skin Tension Lines

## Abstract

Cutaneous defects may result from trauma, infection, chronic illness, poor healing, or surgical resections. Traditionally, the concept of the reconstructive ladder suggests that primary closure and skin grafting should be considered first in reconstruction of such defects. However, these techniques may lead to increased likelihood of dehiscence, distortion of key structures, poor cosmetic outcomes, and less-than-total graft acceptance. To overcome these limitations, various local skin flaps and tissue rearrangement techniques have been developed, including rhomboid flap. This flap is quickly and easily designed, does not require any special instruments, and provides excellent contour, texture, thickness, color match, long-term good cosmesis and high patient satisfaction. The following article presents a comprehensive review of rhomboid flaps in the English literature and discusses the indications, applications, and results. Nearly 100 years after it was first described by A.A. Limberg, the time has come to embrace this simple and elegant flap as the preferred method of reconstruction of cutaneous defects of any size, caused by any etiology and on any part of the body.

## Highlights

• Rhomboid flap is easily designed, quickly executed, without requiring any special instruments.
• It achieves excellent contour, texture, thickness, and color match.
• History, flap design, assessment of defect, indications, and contraindications are discussed.
• The operative technique, caveats, photographs, and review of literature are also included.

## 1 Introduction

The “reconstructive ladder” concept has origins in ancient Egyptian medical texts that were written sometime between 2600 and 2200 BCE. The principle suggests that simplest effective technique should be considered first in reconstruction [1]. However, at times, primary closure or grafting techniques may lead to increased likelihood of dehiscence, distortion of key structures, poor cosmetic outcomes, or less-than-total graft acceptance [2]. In these situations, local flaps with the pliability, matching texture and color become the best option. Among such flaps, a rhomboid flap is a versatile option for reconstruction [3].

In the nearly 100 years since its first description, several refinements have been reported including the “diamond” modification [4], Dufourmental modification with a wider pedicle [5] and Quaba modification to cover circular defects [6]. Rhomboid flaps have been successfully described in head and neck reconstruction, breast reconstruction and pilonidal sinus reconstruction [7]. The indications for use of the rhomboid flap, operative technique, and results in the literature are reviewed here.

## 2 History

The rhomboid flap design was first described by Professor Alexander Alexandrovich Limberg of Leningrad in 1928. The first description in English language was a chapter in *Modern Trends in Plastic Surgery,* edited by Thomas Gibson, in 1963 [8]. The term flap originates from the Dutch word “flappe”, meaning something suspended extensive and loose, attached only by one side, referring to keeping its blood supply by the pedicle [9].

## 3 Flap design

**Fig. 1:**
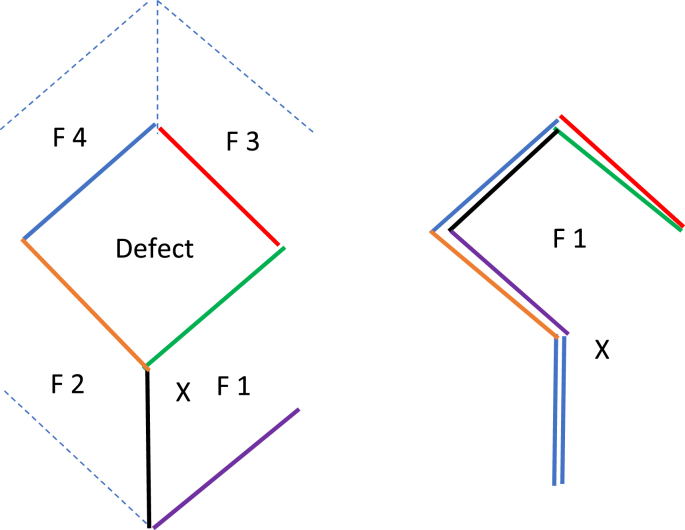
The design is a rhombus with two angles of 120° and two angles of 60°. All sides are equal. A. Several possible flap designs exist for any defect. Four possible flaps, F 2, F 3, F 4 (broken lines), and F 1 (solid lines) are shown. B. Flap F 1 is chosen and rotated across the pivot point, X, superiorly to reconstruct the defect.

The design is a parallelogram with two angles of 120° and two angles of 60°. All sides are equal, and typically four flaps can be raised from one rhomboid (Fig. 1) [8,10]. The rhomboid flap is a flap of skin and subcutaneous tissue that is rotated around a pivot point, X, (Fig. 1) into an adjacent defect [11]. The technique of elevation is simple. The flap's pedicle maintains subpapillary and sub-dermal vascular plexuses to provide superior results when compared to skin grafts of similar size and location [12,13]. Larger sized rhomboid flaps may rely on perforator vascular supply [1,2]. A reduction in tension on the flap decreases the likelihood of necrosis of the donor tissue [14]. The flap should be positioned in the direction of minimal tension and maximum extensibility. Placement of incisions parallel to Relaxed skin tension lines (RSTL) allows the resulting scar to fall within the creases of the skin along line of maximal extensibility and results in a narrower scar [7,[15], [16], [17], [18], [19], [20]].

## 4 Assessment of the defect

A cutaneous defect may be secondary to a trauma, chronic wound, a nonhealing wound, infection, scar formation or a skin lesion removal. It is important to ensure that patient's overall health status is optimal, especially the nutritional parameters and glucose control. Absence of nicotine exposure is ideal. The skin edges should be debrided if needed to achieve good vascularity. The application of this flap has been described in almost all parts of the body with extreme safety and good cosmetic result [1,8]. Traditionally, rhomboid flaps have been safely used to reconstruct small to moderately sized skin defects [2]. However, the authors have presented several cases where very large sized defects have been successfully reconstructed with rhomboid flaps [1,3].

## 5 Indications

Unlike other local flaps, the rhomboid flap can be used in virtually any sized defect and any part of the body. Borges has suggested that in facial reconstructions, flaps are preferable to primary closure and/or grafting even for small lesions [21]. This flap has been described in both genders, all ethnicities and in all the age ranges. It has been widely used in facial, breast, trunk, hand, eyelid, and perianal reconstruction [8]. There is no limitation based on defect etiology, age, or most patient factors [7,8,16]. Cutaneous defect left after tumor resections remains the primary etiologic factor for this flap consideration [7].

Chasmar provided examples of its application in defects of eyelid, floor of nose, alar rim, chin caused by skin cancer, lupus, cystic acne, and spina bifida [8,16]. Alvarez et al. reports the face as the most treated location, followed by the lumbosacral region, dorsal, inguinoscrotal region, thorax, shoulder, and supraclavicular region [16]. Other studies show evidence of success of rhomboid flaps in thigh, upper limb, lower limb, trunk, and pilonidal area reconstruction [[22], [23], [24], [25], [26], [27]]. The familiarity with this technique will likely expand its applicability for every clinician. Its is imperative the procedure should be discussed in detail with each patient, including the size and appearance of potential scar and the typical postoperative course. A signed informed consent should be obtained and documented.

## 6 Contraindications

Although rhomboid flap has been found to be very versatile [1], there are instances in which a different flap design should be considered. The major limitation is in patients with lower body mass index and with less available skin [3]. This is primarily based on relative lack of tissue adjacent to cutaneous defects. For example, for relatively larger nasal defects, a bilobed flap design may be preferable. For reconstructing very large defects, margins of the defect may be mobilized to make the overall size smaller. Additionally, multiple smaller rhomboid flaps can be considered. Other options such as the keystone flap also exist [3]. However, the large defect size is a relative contraindication as the authors have published case reports in the literature where very large trunk defects were successfully reconstructed with rhomboid flap design [1,3]. Patient factors such as nicotine exposure and poorly controlled diabetes are also relative contraindications.

## 7 Room setup

Most Rhomboid flaps may be performed under local anesthesia in an office setting. Intravenous sedation or general anesthesia may be considered in patient with significant comorbidities and cases where more extensive flap procedures are needed.

## 8 Patient markings

**Fig. 2:**
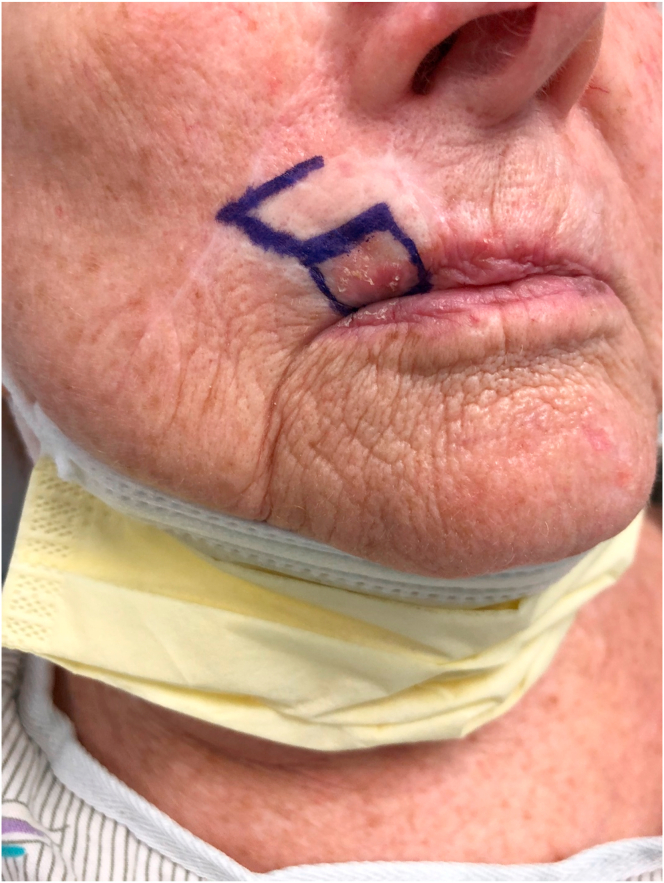
Preoperative view of right upper lip Squamous Cell Cancer showing the marking (in blue) for rhomboid flap. Patient is sitting up with the head is on the top of the photograph. (For interpretation of the references to color in this figure legend, the reader is referred to the Web version of this article.)

The cutaneous primary defect is shaped in the form of a rhombus with four equal sides and with two angles of 120° and two angles of 60° (Fig. 2). Patient may be placed in sitting, supine, lateral or prone position. Typically, four potential flaps can be drawn from one rhomboid defect (Fig. 1) [9]. The size of the adjacent rhomboid flap is similar and maybe slightly smaller than the primary defect (Fig. 2) [3]. For each of the four options, the laxity of the skin is assessed, and the direction of Relaxed Skin Tension Lines (RSTL) is noted. Also, the anatomical position of the key structures is respected to avoid distortion. The final flap option is chosen based on presence of skin laxity and being parallel to RSTL (Fig. 2). In this way, the raised flap rotates to reconstruct the primary defect and the secondary defect is covered by advancement along RSTL. Thus, tension is reduced, and new collagen is oriented in that direction and results in a narrower scar [28]. The goal is to leave patient with best possible aesthetic outcome.

## 9 Operative technique

### 9.1 Anatomy

The cutaneous defect is full-thickness and may be present on any part of the defect. Adjacent tissue is examined, and location of the rhomboid design is based on laxity. The aging process leads to increased laxity. This is a random pattern transposition flap with vascularity based on papillary and sub-dermal vascular plexuses [1]. For larger sized defects, doppler may be used to identify and preserve feeding perforator vessels at the base of the flap and the flap is raised in an axial fasciocutaneous fashion [1,3].

### 9.2 Technique

The patient should be placed in appropriate position. The area to excised should be marked with a rhomboid design and the rhomboid flap be marked adjacent to the defect. The area is prepped and draped in a standard sterile fashion. Vast majority of time, these flaps are performed under local anesthesia, using 1% lidocaine solution with epinephrine 1:100,000. Usually, the level of dissection is relatively superficial maintaining 2–3 mm subcutaneous adipose tissue.

**Fig. 3:**
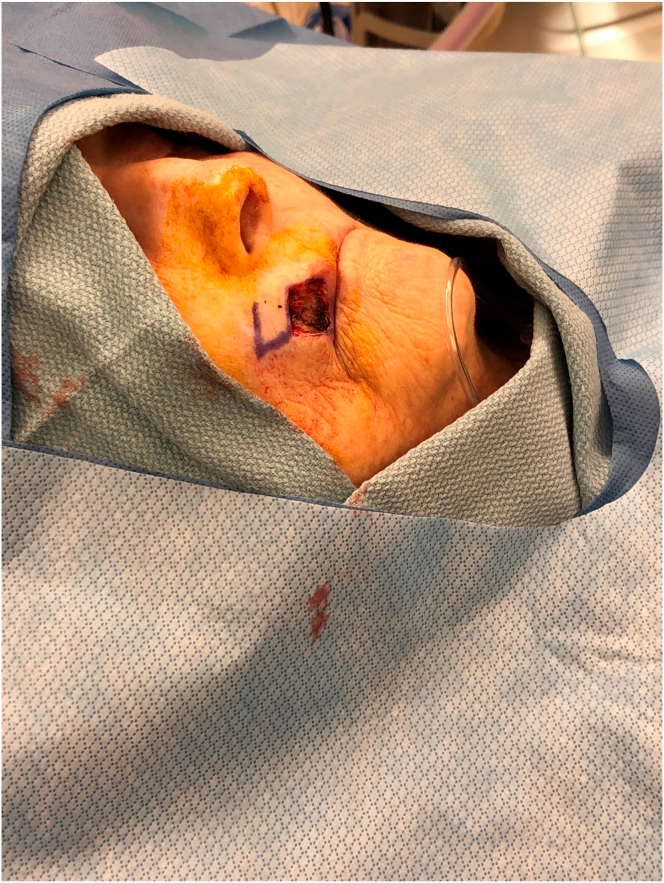
Intraoperative view of defect left after excision of Squamous cell cancer. Rhomboid flap is marked superior and lateral to defect. Patient is in supine position. The head is on the right side and feet on the left of the photograph.

**Fig. 4:**
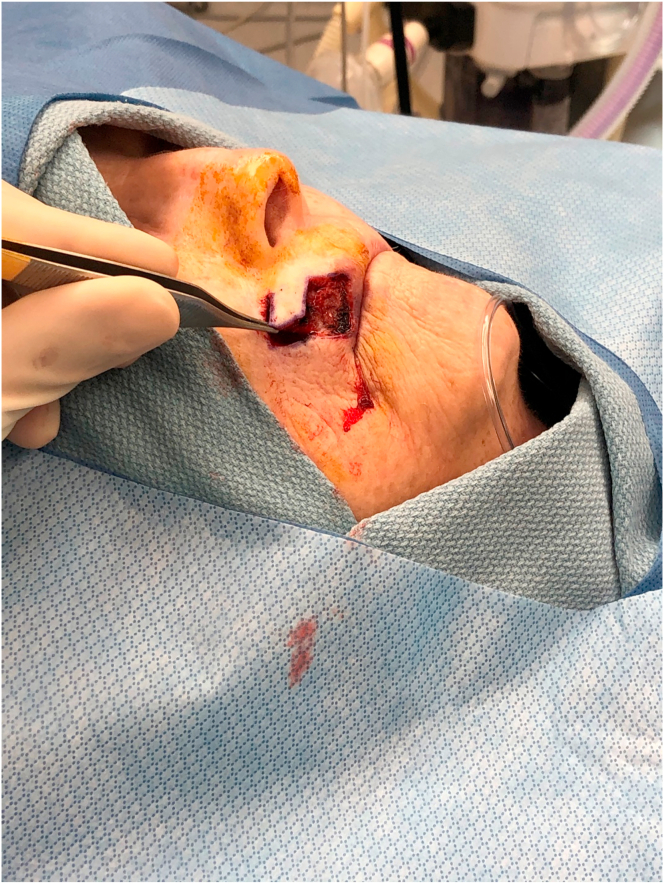
Intraoperative view of beginning of Rhomboid flap elevation. The tip of forceps is holding the flap. Patient is in supine position. The head is on the right side and feet on the left of the photograph.

**Fig. 5:**
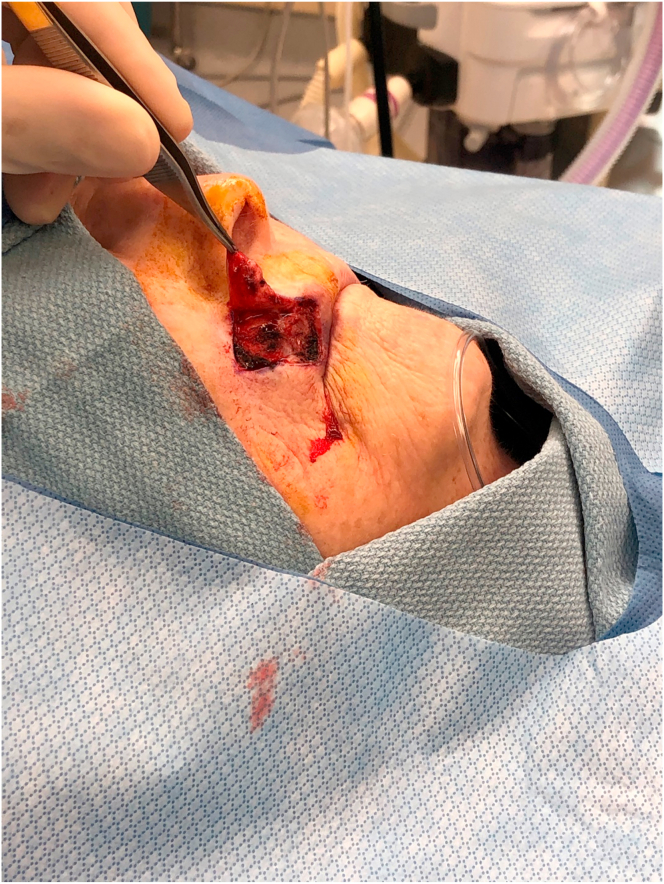
Intraoperative view of completion of Rhomboid flap elevation. The tip of forceps is holding the flap. Patient is in supine position. The head is on the right side and feet on the left of the photograph.

**Fig. 6:**
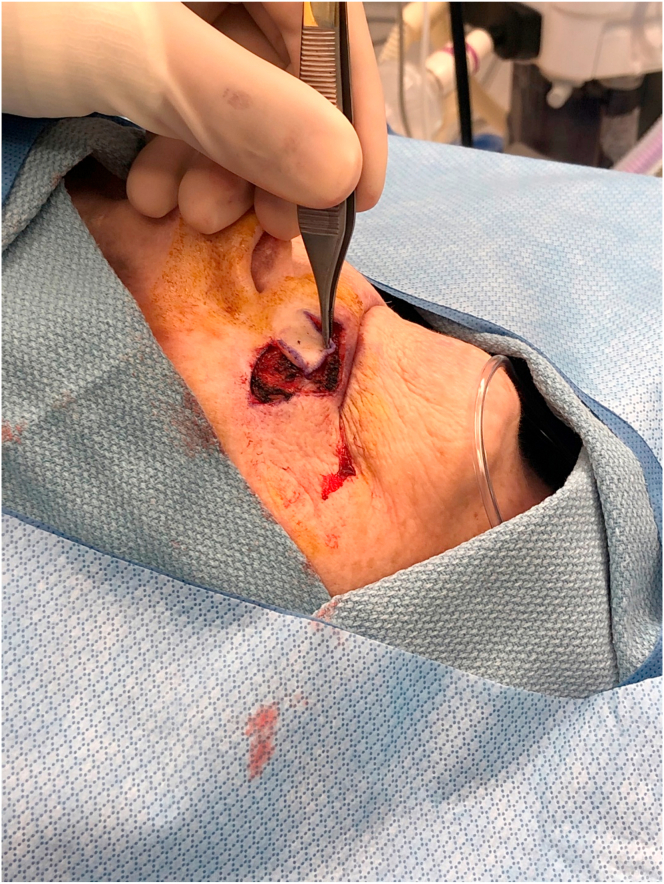
Intraoperative view of Rhomboid flap rotation medially and inferiorly to obliterate the defect. The tip of forceps is holding the flap. Patient is in supine position. The head is on the right side and feet on the left of the photograph.

**Fig. 7:**
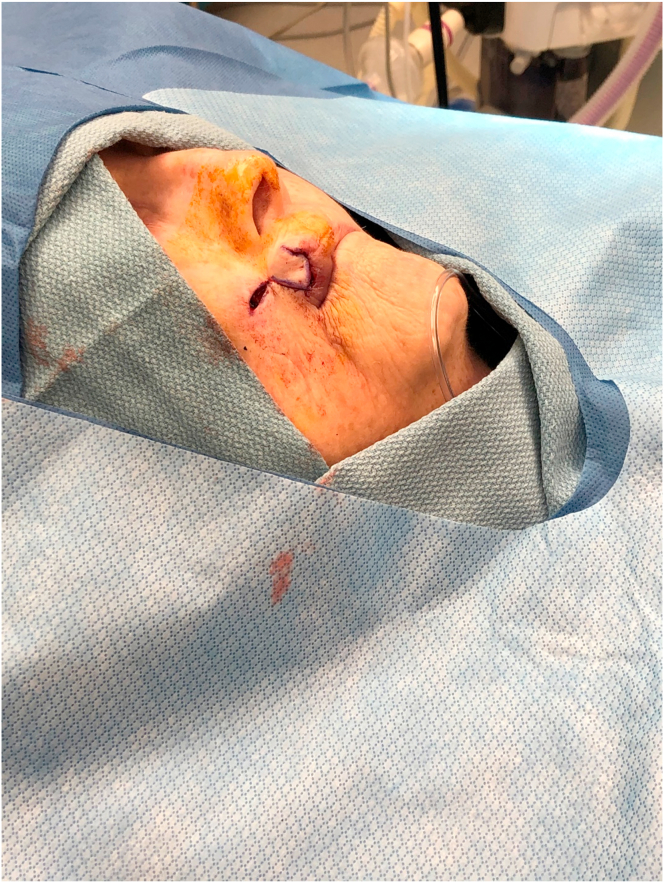
Intraoperative view of Rhomboid flap inset with deep sutures to obliterate the defect. Patient is in supine position. The head is on the right side and feet on the left of the photograph.

**Fig. 8:**
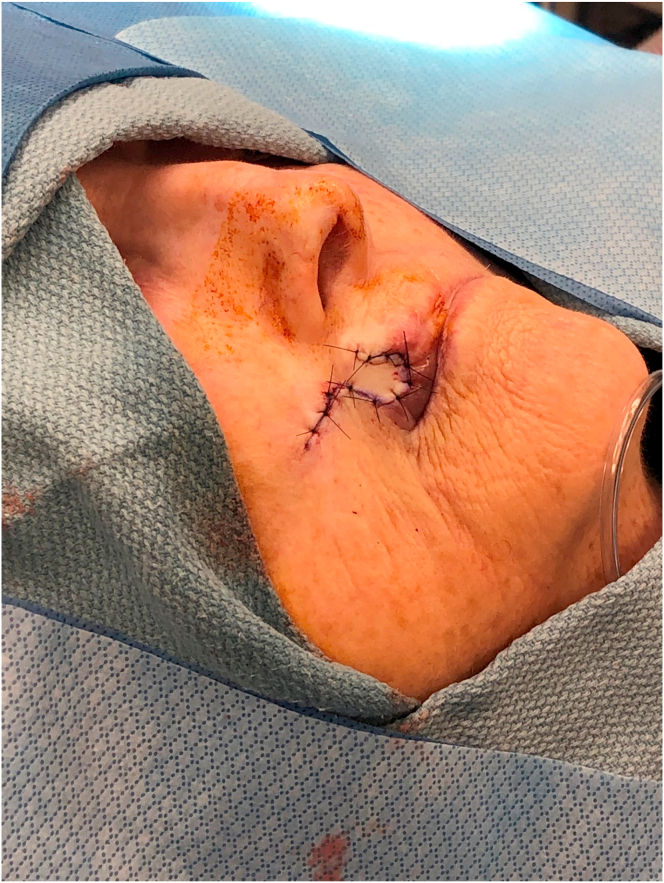
Intraoperative view of Rhomboid flap inset with superficial sutures to obliterate the defect. Patient is in supine position. The head is on the right side and feet on the left of the photograph.

A76-year-old-female presented with a 1.0 cm diameter biopsy proven Squamous Cell Cancer of right upper lip (Fig. 2). Under intravenous anesthesia, the 1 cm × 1 cm area with margins was marked to be excised. A rhomboid flap was marked along the lateral superior aspect of the defect because of the maximum skin laxity and resting skin tension lines (Fig. 3). The rhomboid flap was raised at the level of fascia (Fig. 4, Fig. 5). The flap was rotated inferiorly and medially to obliterate the defect (Fig. 6) and inset using numerous 4–0 Biosyn interrupted sutures (Fig. 7) and 5-0 Nylon sutures (Fig. 8). Both primary and secondary defects were obliterated under acceptable tension and in a single stage.

## 10 Postoperative management

Postoperatively, the patient may be instructed to use antibiotic ointment twice daily along sutures. Skin sutures are generally removed in 1–2 weeks based on the location. Usually, the patients do not have any restrictions and heal within a few days.

## 11 Caveats

A well-planned rhomboid flap maintains continuity of texture, color, thickness, and vascularity with the surrounding tissue, eliciting the most successful functional and aesthetic outcome [16,18,19]. The technique of its elevation is simple. Its minimally invasive, quick to perform, has reduced tension, rapid healing and requires a single stage under local anesthesia [19]. The resultant scar is geometric broken lines that is less noticeable than longer linear primary closures [11,12,29] and the secondary defect scar can be hidden in a RSTL, making it less apparent [11,19]. This flap design can be used to close defects almost anywhere on the body, making this a preferred technique [8].

## 12 Rhomboid and local flaps in the literature

Numerous reports in the literature support using rhomboid flap for successful closure of cutaneous defects, in different anatomical locations, with high patient satisfaction and low complications.

A single institution experience in 70 patients treated for facial malignancies, revealed local flaps gave the best results and were the first choice of reconstruction of the face. The study acknowledges that smooth contour and scar quality are very important for Plastic Surgery patients [26,[30], [31], [32]].

Li et al. reported a series of 41 satisfied patients out of 48 who underwent excision of benign pigmented nevi, and then underwent local flap reconstruction of defects ranging from 0.9 cm to 11 cm [33]. Another series reports 100% satisfaction in 21 patients who underwent local flap reconstruction after excision of benign lesions of less than 1 cm defects [33]. A series of 27 patients who underwent a medial canthal reconstruction with a rhomboid flap showed healing with no major complications and re-operations [11].

Alvarez et al. reports a series of 50 patients where face was the location 72% of the time with very few complications [16]. Another series of 30 patients of rhomboid flaps healed without any significant complications [14]. A series of 35 flaps for reconstruction after cutaneous malignancy resection, revealed excellent outcomes [31]. Tissiani et al. published a series of 45 double transposition flaps, where 70% defects were small, with a 15% complication rate [24]. A series of 175 reconstructions reported a complication rate of 9.1%, including infection, hematoma, partial necrosis, and partial dehiscence [6].

The only landmark article for comparison of rhomboid flap and primary closure was published as a meta-analysis of randomized controlled trials. The authors used the defect left behind after excision of sacrococcygeal pilonidal disease. 641 patients were included with rhomboid flaps demonstrating statistically significant trend towards lower wound infection and dehiscence. The conclusion was that the rhomboid flap was superior to primary closure [34].

The traditional teaching involves a combination of direct teaching from experts and text-based learning. However, the relatively easy learning curve for rhomboid flap made it the flap of choice for a Brazilian study comparing computer assisted training versus standard text-based education of medical trainees. The study concluded that computer assisted training resulted in better performance and was ranked best teaching tool [35].

## 13 Conclusion

Every case should be approached in an individual manner as no two patients, nor two defects are the same.^1^ Reconstruction should be tailored to the unique characteristics of the defect, patient expectations and surgeon experience.^2^ We believe that with proper patient selection, rhomboid flaps should be considered a first-line option for reconstruction of almost any defect caused by any etiology. In summary, the technical ease, aesthetic outcome, continuity of function, short operation time, matching skin texture and color, safety, early functionality, tension-free closure, and fewer out-patient clinic visits are some of the reasons to justify extensive applications of rhomboid flaps.

## Patient/guardian consent

The patient has given consent for possible publication of the photographs for this review.

## Ethical approval

No Institutional Review Board approval needed for review at our institution.

## Sources of funding

No sponsor and no funding.

## Consent

Written informed consent was obtained from the patient for publication of this review and accompanying images. A copy of the written consent is available for review by the Editor-in-Chief of this journal on request.

## Author contribution

AK, senior author, performed the intervention. Both authors, AK and KK, contributed to manuscript. Both authors have read and agreed with the manuscript.

## Trial registry number

1. Name of the registry: NA. This is a case report of one patient.
2. Unique Identifying number or registration ID:
3. Hyperlink to your specific registration (must be publicly accessible and will be checked):

## Declaration of competing interest

No conflict of interest from both authors.
